# Association of spirometric restriction with mortality in the silicotics: a cohort study

**DOI:** 10.1186/s12890-023-02622-1

**Published:** 2023-09-04

**Authors:** Shuyuan Yang, Chi Kuen Chan, Maggie Haitian Wang, Chi Chiu Leung, Lai Bun Tai, Lap Ah Tse

**Affiliations:** 1 Jockey Club School of Public Health and Primary Care, The Chinese University of Hong Kong, Hong Kong SAR, China; 2 Tuberculosis and Chest Service, Department of Health, Hong Kong SAR, China; 3 Stanley Ho Centre for Emerging Infectious Diseases, The Chinese University of Hong Kong, Hong Kong SAR, China; 4 Institute of Space and Earth Information Science, The Chinese University of Hong Kong, Hong Kong SAR, China

**Keywords:** Lung function, Restrictive spirometry pattern, Airflow obstruction, Mortality, Silicosis

## Abstract

**Background:**

Restrictive spirometry pattern (RSP), defined as reduced forced vital capacity (FVC) in absence of airflow obstruction (AFO), is associated with increased risk of mortality in general population. However, evidence in the patients with silicosis is limited. This study was aimed to investigate the relationship between RSP and the risk of death in a silicotic cohort.

**Method:**

This retrospective cohort study used data from the Pneumoconiosis Clinic, Hong Kong Department of Health that containing 4315 patients aged 18–80 years and diagnosed with silicosis during 1981–2019, with a follow-up till 31 December 2019. Spirometry was carried out at the diagnostic examination of silicosis. Lung function categories were classified as normal spirometry (FEV_1_/FVC ≥ 0.7, FVC ≥ 80% predicted), RSP only (FEV_1_/FVC ≥ 0.7, FVC < 80% predicted), AFO only (FEV1/FVC < 0.7, FVC ≥ 80% predicted), and RSP&AFO mixed (FEV_1_/FVC < 0.7, FVC < 80% predicted). The hazard ratio (HR) and 95% confidence intervals (95% CI) were computed using a Cox proportional hazards model adjusting for age, body mass index, history of tuberculosis, smoking status, pack-years, and radiographic characteristics of silicotic nodules.

**Results:**

Among the 4315 patients enrolled in the study, the prevalence of RSP was 24.1% (n = 1038), including 11.0% (n = 473) with RSP only and 13.1% (n = 565) with mixed RSP and AFO. During the follow-up period, a total of 2399 (55.6%) deaths were observed. Compared with the silicotics with normal spirometry, those with RSP only had significantly increased risk of all-cause mortality (HR = 1.63, 95% CI 1.44–1.85) and respiratory-related mortality (HR = 1.56, 95% CI 1.31–1.85). Notably, a higher risk of mortality was observed in silicotics with mixed ventilatory defects of both RSP and AFO (all-cause mortality: HR = 2.22, 95% CI 1.95–2.52; respiratory-related mortality: HR = 2.59, 95% CI 2.18–3.07) than in those with RSP only.

**Conclusion:**

RSP is significantly associated with increased risk of all-cause and respiratory-related mortality in the silicotics, and patients with mixed restrictive and obstructive ventilatory defect have higher risk of mortality than those with single RSP or AFO. These findings emphasize the importance of recognizing RSP in the occupational settings, especially for the silicotic patients with mixed ventilatory defect.

**Supplementary Information:**

The online version contains supplementary material available at 10.1186/s12890-023-02622-1.

## Background

Silicosis is one of the most important occupational diseases characterized by pulmonary fibrosis due to inhalation of respirable crystalline silica which threatens over 30 million workers around the world [1, 2]. In recent years, failure to recognize and eliminate the silica-related exposure in some contemporary work practices including denim jean production, domestic benchtop fabrication and jewellery polishing have led to a global re-emergence of this irreversible interstitial lung disease [3, 4]. Spirometric restriction, alternatively termed as restrictive spirometry pattern (RSP) and defined as a reduced forced vital capacity (FVC) in absence of airflow obstruction (AFO), is associated with increased morbidity and mortality [5]. Recently, a growing body of literature based in general population showed that RSP was associated with poor quality of life [6], increased comorbidities (e.g., cardiovascular events [7], diabetes mellitus [8], and metabolic syndrome [9]) and all-cause mortality [10]. Despite a prevalence of around 14.2% worldwide [11, 12], this abnormal spirometry phenotype is often underdiagnosed and thus untreated in the clinical settings currently due to the lack of established guidelines [13]. Although RSP does not necessarily indicate true lung restriction and may result from adult obesity or other lung conditions such as air trapping that also presented as low FVC [14, 15], its significant association with adverse health outcomes implies that RSP, as an abnormal spirometry phenotype, might serve as a promising marker for clinicians to better predict the health status of patients and thereafter provide timely intervention.

Higher prevalence of RSP was observed in workers with silicosis than in the general population [16]. Although significant associations of RSP with all-cause, respiratory-related and cardiovascular-related mortality have been reported in several population-based studies [10, 17–22], there is limited evidence on whether and to what extent of fibrotic changes in lungs contributes to and interacts with RSP to have impact on disease prognosis. Besides, AFO has been taken into consideration in the assessment of silicosis compensation claims because of its well-understood clinical prognosis [23], but less is clear for the prognostic value of RSP as another abnormal spirometry phenotype to predict the risk of mortality, particularly among smoking silicotics. Therefore, the present study aimed to depict a whole picture of abnormal spirometry phenotype in the form of RSP and AFO and then determine their associations with all-cause and cause-specific mortality in a large occupational cohort of 4315 workers with confirmed silicosis.

## Methods

### Cohort enumeration and data

This is a retrospective cohort study of a territory-wide silicosis cohort including consecutive patients diagnosed with silicosis in Hong Kong during 1981–2019. The details of this cohort have been described elsewhere [24–26]. Briefly, this cohort contains 4481 workers diagnosed with silicosis at the Pneumoconiosis Clinic, Tuberculosis and Chest Service of Department of Health since 1 January 1981 and followed up until 31 December 2019. Silicosis was diagnosed based on the radiographic changes in combination with a work history involving occupational exposure to silica-related dusts [27]. During the diagnostic evaluation, three members of the Pneumoconiosis Medical Board independently viewed chest X-ray films with the International Labor Office (ILO) criteria of presence of round and/or irregular lung opacities with profusion equal to or greater than subcategory 1/0 according to the International Classification of Radiographs of Pneumoconiosis [28]. Each worker’s lifetime work history, lifelong smoking habits and history of tuberculosis were extracted from the medical records at the Pneumoconiosis Clinic. The industry and nature of job were classified using the Statistical Classification of Economic Activities in the European Community Rev. 2 (2008): Level 1 Codes, and the silica-exposed job tasks were categorised as stone cutter, bricklayer, labourer, masonry worker, foreman, tuck pointer, grinder, hod carrier, quarryman, jeweller, and decoration worker. Cumulative silica exposure was estimated using a job-exposure matrix (JEM) based on the exposure levels summarized by the Occupational Safety and Health Administration (OSHA) [29]. After linking the individual occupation to the job-exposure matrix, the estimated silica dust exposure for each episode of job can be calculated by multiplying the exposure level of the certain job by job duration. The cumulative dust exposure (mg/m^3^-year) was obtained by summing up the exposure of all episodes of jobs, as in Eq. (1).

1 $$Cumulative\,silica\,exposure = \sum\limits_{{\rm{i = 1}}}^n {{E_i}*{T_i}}$$

*where E*_*i*_ *= the geometric mean of crystalline silica exposure for the i* ^*th*^ *episode of job; T*_*i*_ *= the net years of crystalline silica exposure for the i* ^*th*^ *episode of job.*

This study was approved by the Survey and Behavior Research Ethics Committee of the Chinese University of Hong Kong (Reference No. SBRE-19-023).

### Spirometry

Spirometry was performed during the pneumoconiosis compensation assessment at the diagnosis of silicosis, whereas such compensation was entitled to all workers with confirmed silicosis no matter whether they have any incapacity of their lung function. Lung function parameters, including forced expiratory volume in one second (FEV_1_) and forced vital capacity (FVC), were measured using a wedge-type bellow spirometer (Vitalograph PFT II plus, Buckingham, UK), with the results corrected for body temperature, water vapor saturation and pressure. The ATS guidelines were followed to ensure the validity and reproducibility of spirometry. Three readings of FEV_1_ and FVC from satisfactory maneuvers were recorded, and only the best was used in analyses. The predicted values and lower limit of normal (LLN) of FEV_1_ and FVC were calculated using the reference equations developed by the Hong Kong Thoracic Society for the local population [30, 31]. The lung function was categorized as: (1) normal spirometry: FEV_1_/FVC ≥ 0.70, FVC ≥ 80% predicted; (2) RSP only: FEV_1_/FVC ≥ 0.70, FVC ≤ 80% predicted; (3) AFO only: FEV_1_/FVC < 0.70, FVC ≥ 80% predicted; (4) RSP&AFO mixed: FEV_1_/FVC < 0.70, FVC < 80% predicted. In the sensitivity analyses, the lung function was also categorized using LLN as (1) normal spirometry: FEV_1_/FVC ≥ LLN, FVC ≥ LLN; (2) RSP only: FEV_1_/FVC ≥ LLN, FVC ≤ LLN; (3) AFO only: FEV_1_/FVC < LLN, FVC ≥ LLN; (4) RSP&AFO mixed: FEV_1_/FVC < LLN, FVC < LLN.

### Clinical outcomes

All subjects with confirmed silicosis were follow-up until the study end date, i.e., 31 December 2019. The median time between baseline spirometry and death or the study end day was 12.3 years, with an interquartile range from 5.9 to 19.4 years. When a subject died, the detailed mortality data including the date and underlying cause of death were extracted from the official death certificate issued by a registered medical partitioner who attended this subject’s last illness. The underlying and contributing causes of death were coded according to the International Statistical Classification of Diseases and Related Health Problem, 10th Revision (ICD-10). The outcomes of this study were (1) all-cause mortality (A00-Z99); (2) respiratory-related mortality (J00-J99); (3) lung cancer mortality (C34); and (4) cardiovascular-related mortality (I00-I99).

### Statistical analyses

Univariate comparisons between subjects with different lung function categories were made using Chi-square tests (categorical), t-tests (parametrical), and Mann-Whitney U tests (non-parametrical). The cumulative survival curves were constructed using Kaplan-Meier method and compared by log-rank test. Overall survival by lung function category was examined using Cox proportional hazard models adjusting for age, body mass index, history of tuberculosis, cumulative silica exposure, smoking status, pack-years, and radiographic changes, i.e., size, profusion of small opacities and progressive massive fibrosis. The risk of cause-specific deaths was assessed using the Fine-Gray competing risk model [32]. The association between FVC % predicted and radiographic changes was examined using a linear regression model. In subgroup analyses, the risk of all-cause mortality was reassessed in subjects with different shape, size, or profusion of silicotic nodules using cox proportional hazard models with the same adjustments as the main analyses. In the sensitivity analyses, a new spirometry pattern (non-RSP preserved ratio impaired spirometry, non-RSP PRISm), defined as FEV_1_ < 80% predicted with normal FEV_1_/FVC ratio and FVC, was subdivided from normal spirometry group and included in the Cox regression to determine its mortality risk. To ensure the robustness of results, the key analyses were carried out based on LLN-defined lung function categories. All statistical analyses were carried out using SAS version 9.4 (SAS Institute, Cary, North Carolina). A two-side *p*-value less than 0.05 was considered as statistically significant.

## Results

### Cohort characteristics and prevalence of RSP

A total of 4481 subjects were diagnosed with silicosis at the Pneumoconiosis Clinic during the study period, of whom 166 (3.7%) were excluded for aged over 80 years old (due to the limitation of prediction formulae for reference values), without baseline physical examination or valid spirometry results (Table E1). Among the remaining 4315 subjects included in the current study, the mean age at baseline was 56.2 (± 10.2) years and 99.4% were males. The prevalence of RSP detected at the baseline medical examination was 24.1% (n = 1038), including 11.0% (n = 473) with RSP only and 13.1% (n = 565) with mixed RSP and AFO. Compared with subjects with normal spirometry, those with RSP only had similar ever smoking rate (85% vs. 86.5%), cumulative silica exposure (1.76 mg/m^3^·year vs. 1.74 mg/m^3^·year) and exposure duration (22.9 years vs. 22.5 years) but more mean pack-years (24.9 vs. 21.1), more advanced fibrotic changes (size of opacities, r or u: 12.4% vs. 5.0%; profusion of opacities, category 3: 18.8% vs. 5.1%; progressive massive fibrosis: 24.9% vs. 12.3%), and higher proportions of underweight (15.4% vs. 4.1%) and history of tuberculosis (53.7% vs. 38.1%), whereas subjects with mixed RSP and AFO had the heaviest smoking pack-years (32.3) and the highest proportions of underweight (22.7%) and ever tuberculosis (57.5%) **(**Table 1**)**. The FVC % predicted was significantly associated with the size and profusion of small lung opacities and progressive massive fibrosis (*p* < 0.001).

**Table 1:** Baseline characteristics of the cohort (n = 4315) by lung function categories

Characteristics	Total	Lung function categories				
		Normal spirometry	RSP only	AFO only	RSP&AFO mixed	*p*-value
Subjects No. (%)	4315 (100.0)	2129 (49.3)	473 (11.0)	1148 (26.6)	565 (13.1)	
Age						
18–34	36 (0.8)	28 (1.3)	2 (0.4)	1 (0.1)	5 (0.9)	< 0.001
35–49	1215 (28.2)	814 (38.2)	146 (30.9)	183 (15.9)	72 (12.7)	
50–64	2140 (49.6)	1013 (47.6)	232 (49.0)	607 (52.9)	288 (51.0)	
≥ 65	924 (21.4)	274 (12.9)	93 (19.7)	357 (31.1)	200 (35.4)	
Sex						
Male	4287 (99.4)	2118 (99.5)	467 (98.7)	1145 (99.7)	557 (98.6)	0.01
Female	28 (0.6)	11 (0.5)	6 (1.3)	3 (0.3)	8 (1.4)	
BMI categories						
Underweight (< 18.5)	371 (8.6)	87 (4.1)	73 (15.4)	83 (7.2)	128 (22.7)	< 0.001
Normal (18.5–22.9)	2132 (49.4)	1016 (47.7)	227 (48.0)	623 (54.3)	266 (47.1)	
Overweight (23-24.9)	942 (21.8)	530 (24.9)	80 (16.9)	251 (21.9)	81 (14.3)	
Obese (≥ 25)	870 (20.2)	496 (23.3)	93 (19.7)	191 (16.6)	90 (15.9)	
Smoking status						
Never smoker	493 (11.4)	288 (13.5)	71 (15.0)	78 (6.8)	56 (9.9)	< 0.001
Current smoker	1965 (45.5)	1018 (47.8)	181 (38.3)	569 (49.6)	197 (34.9)	
Former smoker	1857 (43.0)	823 (38.7)	221 (46.7)	501 (43.6)	312 (55.2)	
Pack-years						
Never smoker	493 (11.5)	288 (13.6)	71 (15.0)	78 (6.8)	56 (10.0)	< 0.001
Below 20	1455 (33.9)	820 (38.7)	151 (31.9)	338 (29.6)	146 (26.0)	
20 to 39	1408 (32.8)	675 (31.8)	158 (33.4)	405 (35.5)	170 (30.2)	
40 or above	942 (21.9)	338 (15.9)	93 (19.7)	321 (28.1)	190 (33.8)	
History of tuberculosis						
Yes	1951 (45.2)	812 (38.1)	254 (53.7)	560 (48.8)	325 (57.5)	< 0.001
No	2364 (54.8)	1317 (61.9)	219 (46.3)	588 (51.2)	240 (42.5)	
Shape of small nodules						
Round	2873 (67.3)	1483 (70.3)	290 (62.2)	802 (70.5)	298 (54.0)	< 0.001
Irregular	1393 (32.7)	628 (29.7)	176 (37.8)	335 (29.5)	254 (46.0)	
Size of small nodules						
Category p or s	1796 (42.1)	984 (46.6)	152 (32.6)	485 (42.7)	175 (31.7)	< 0.001
Category q or t	2092 (49.0)	1022 (48.4)	256 (54.9)	531 (46.7)	283 (51.3)	
Category r or u	378 (8.9)	105 (5.0)	58 (12.4)	121 (10.6)	94 (17.0)	
Profusion of small nodules						
Category 1 (1/0, 1/1, 1/2)	2384 (55.9)	1284 (60.9)	162 (34.7)	674 (59.4)	264 (48.0)	< 0.001
Category 2 (2/1, 2/2, 2/3)	1528 (35.9)	719 (34.1)	217 (46.5)	382 (33.7)	210 (38.2)	
Category 3 (3/2, 3/3, 3/+)	350 (8.2)	107 (5.1)	88 (18.8)	79 (7.0)	76 (13.8)	
Progressive massive fibrosis						
No (small opacities only)	3516 (82.2)	1853 (87.7)	352 (75.1)	900 (78.9)	411 (73.8)	< 0.001
Yes (with large opacity)	763 (17.8)	259 (12.3)	117 (24.9)	241 (21.1)	146 (26.2)	
Respiratory symptoms						
Cough	2903 (67.3)	1386 (65.1)	303 (64.3)	806 (70.3)	408 (72.2)	< 0.001
Dyspnoea	3366 (78.0)	1536 (72.1)	387 (82.0)	931 (81.2)	512 (90.6)	< 0.001
Sputum	2540 (58.9)	1176 (55.2)	252 (53.4)	734 (64.0)	378 (66.9)	< 0.001
Chest pain	1444 (33.5)	807 (37.9)	134 (28.4)	359 (31.3)	144 (25.5)	< 0.001
Wheeze	738 (17.1)	253 (11.9)	58 (12.3)	287 (25.0)	140 (24.8)	< 0.001
Haemoptysis	416 (9.7)	179 (8.4)	43 (9.1)	125 (10.9)	69 (12.2)	0.02
Duration of silica exposure (year)	23.5 (11.3)	22.5 (10.9)	22.9 (11.5)	25.5 (11.2)	23.8 (11.9)	< 0.001
Cumulative silica exposure (mg/m^3^·year)	1.80 (0.86)	1.74 (0.84)	1.76 (0.89)	1.93 (0.85)	1.80 (0.91)	< 0.001

Abbreviations: AFO, airflow obstruction; RSP, restrictive spirometry pattern; BMI, body mass index

Data are presented as n (raw %), n (column %), n (% yes), or mean (SD)

### Mortality by lung function category

**Fig. 1:**
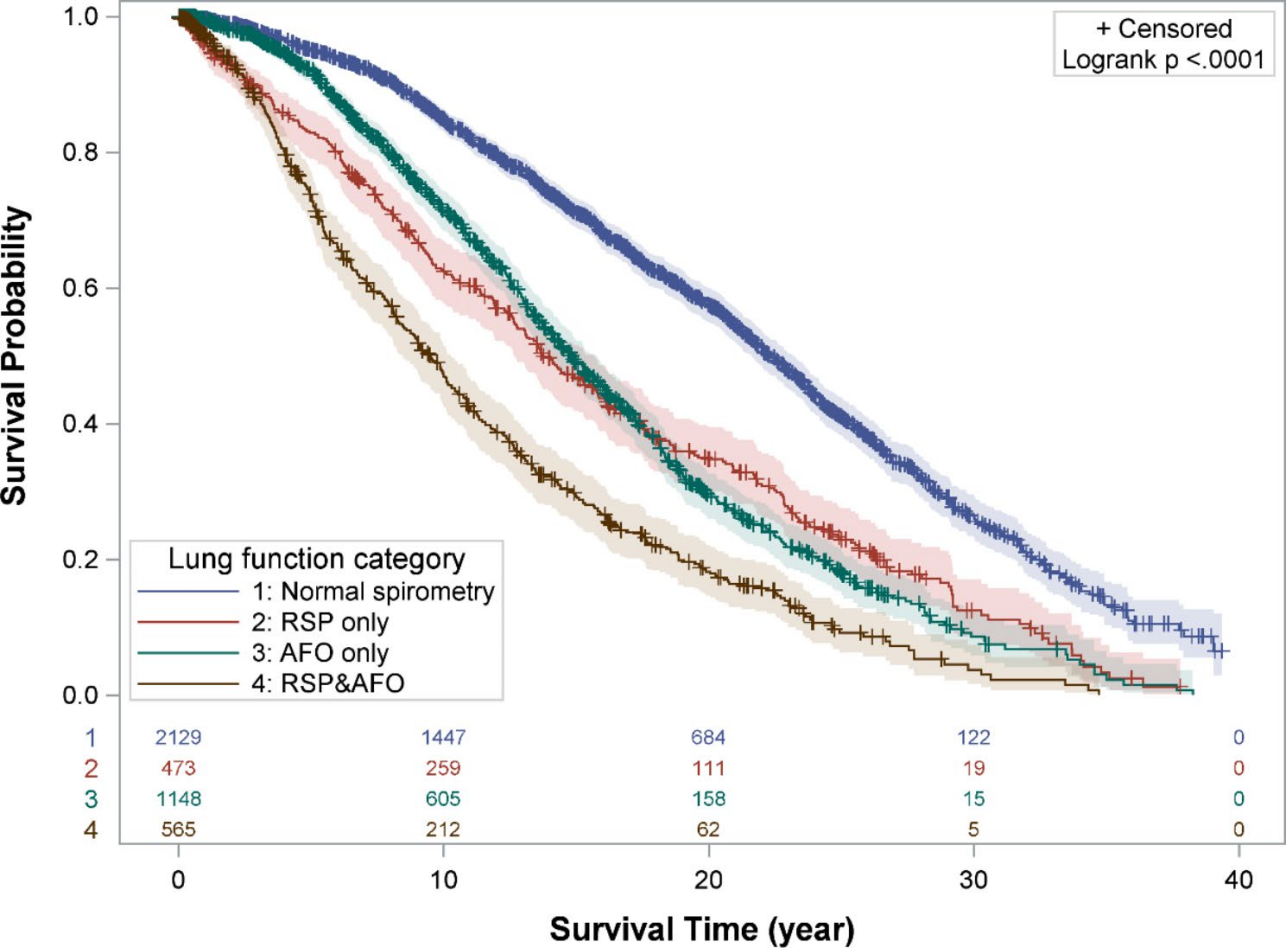
Kaplan-Meier curves of all-cause mortality with 95% confidence interval by baseline lung function categories Abbreviations: RSP, restrictive spirometry pattern; AFO, airflow obstruction

During the study period, 2399 (55.6%) deaths were observed among the 4315 subjects. The leading causes of death were the diseases of the respiratory system (J00-J99) (1359 subjects, 56.6%), malignant neoplasms (C00-C97) (513 subjects, 21.4%) and diseases of the circulatory system (I00-I99) (168 subjects, 7.0%) (Table E2). Subjects with RSP only had significantly lower survival probability than those with normal spirometry but the probabiliy was higher than those with mixed RSP and AFO **(**Fig. 1**)**. Compared with the subjects with normal spirometry, those with RSP only or RSP&AFO mixed had significantly increased risk of all-cause mortality (crude HR = 1.91, 95% CI 1.69–2.16 for RSP only; crude HR = 3.31, 95% CI 2.95–3.72 for RSP&AFO mixed) and respiratory-related mortality (crude HR = 1.88, 95% CI 1.59–2.22 for RSP only; crude HR = 3.38, 95% CI 2.90–3.94 for RSP&AFO mixed), and these associations remained significant after adjusting for age, BMI, tuberculosis, cumulative silica exposure, smoking habits and radiographic changes (all-cause mortality: adjusted HR = 1.63, 95% CI 1.44–1.85 for RSP only, adjusted HR = 2.22, 95% CI 1.95–2.52 for RSP&AFO mixed; respiratory-related mortality: adjusted HR = 1.56, 95% CI 1.31–1.85 for RSP only, adjusted HR = 2.59, 95% CI 2.18–3.07 for RSP&AFO mixed) **(**Table 2**)**. There was no significant association between RSP, including both RSP only and RSP&AFO mixed, and the risk of mortality from cardiovascular-related diseases and lung cancer.

**Table 2:** Hazard ratios (HR) and 95% confidence intervals (CI) for all-cause and major cause-specific mortality by lung function categories

Lung function categories	No. of deaths/ subjects	Crude mortality rate*	Crude HR (95% CI)	Adjusted HR (95% CI)		
			Model 0	Model 1	Model 2	Model 3
All-cause mortality						
Normal spirometry	991/2129	30.7	1.00 (Ref.)	1.00 (Ref.)	1.00 (Ref.)	1.00 (Ref.)
RSP only	335/473	55.5	1.91 (1.69, 2.16)	1.75 (1.54, 1.98)	1.76 (1.55, 1.99)	1.63 (1.44, 1.85)
AFO only	662/1148	49.6	1.93 (1.75, 2.14)	1.55 (1.40, 1.72)	1.50 (1.35, 1.67)	1.47 (1.32, 1.63)
RSP&AFO mixed	411/565	80.0	3.31 (2.95, 3.72)	2.50 (2.21, 2.82)	2.37 (2.10, 2.69)	2.22 (1.95, 2.52)
Respiratory-related mortality						
Normal spirometry	499/2129	15.4	1.00 (Ref.)	1.00 (Ref.)	1.00 (Ref.)	1.00 (Ref.)
RSP only	193/473	32.0	1.88 (1.59, 2.22)	1.76 (1.48, 2.08)	1.76 (1.49, 2.08)	1.56 (1.31, 1.85)
AFO only	379/1148	28.4	1.77 (1.55, 2.01)	1.62 (1.42, 1.86)	1.61 (1.40, 1.85)	1.55 (1.34, 1.78)
RSP&AFO mixed	288/565	56.1	3.38 (2.90, 3.94)	2.94 (2.51, 3.45)	2.89 (2.46, 3.40)	2.59 (2.18, 3.07)
Lung cancer mortality						
Normal spirometry	127/2129	3.9	1.00 (Ref.)	1.00 (Ref.)	1.00 (Ref.)	1.00 (Ref.)
RSP only	29/473	4.8	0.96 (0.64, 1.44)	0.91 (0.60, 1.38)	0.96 (0.64, 1.46)	0.92 (0.60, 1.41)
AFO only	73/1148	5.5	1.14 (0.86, 1.52)	0.95 (0.70, 1.28)	0.86 (0.63, 1.16)	0.85 (0.63, 1.17)
RSP&AFO mixed	20/565	3.9	0.60 (0.37, 0.96)	0.49 (0.30, 0.80)	0.49 (0.30, 0.80)	0.48 (0.29, 0.81)
Cardiovascular-related mortality						
Normal spirometry	78/2129	2.4	1.00 (Ref.)	1.00 (Ref.)	1.00 (Ref.)	1.00 (Ref.)
RSP only	27/473	4.5	1.44 (0.93, 2.23)	1.43 (0.91, 2.24)	1.41 (0.90, 2.23)	1.47 (0.94, 2.31)
AFO only	37/1148	2.8	0.95 (0.64, 1.40)	0.80 (0.53, 1.20)	0.80 (0.53, 1.21)	0.86 (0.57, 1.31)
RSP&AFO mixed	26/565	5.1	1.29 (0.83, 2.02)	1.10 (0.68, 1.79)	1.12 (0.68, 1.83)	1.14 (0.68, 1.91)

Abbreviations: AFO, airflow obstruction; RSP, restrictive spirometry pattern; HR, hazard ratio; CI, confidence interval

Model 0: no adjustments

Model 1: adjusted for age, BMI category, history of tuberculosis, and cumulative silica exposure

Model 2: adjusted for the covariates in Model 1 plus smoking status and pack-years

Model 3: adjusted for the covariates in Model 2 plus the radiographic characteristics of the silicotic nodules, including shape, size, profusion of the small opacities and progressive massive fibrosis

* Per 10^3^ person-years

**Fig. 2:**
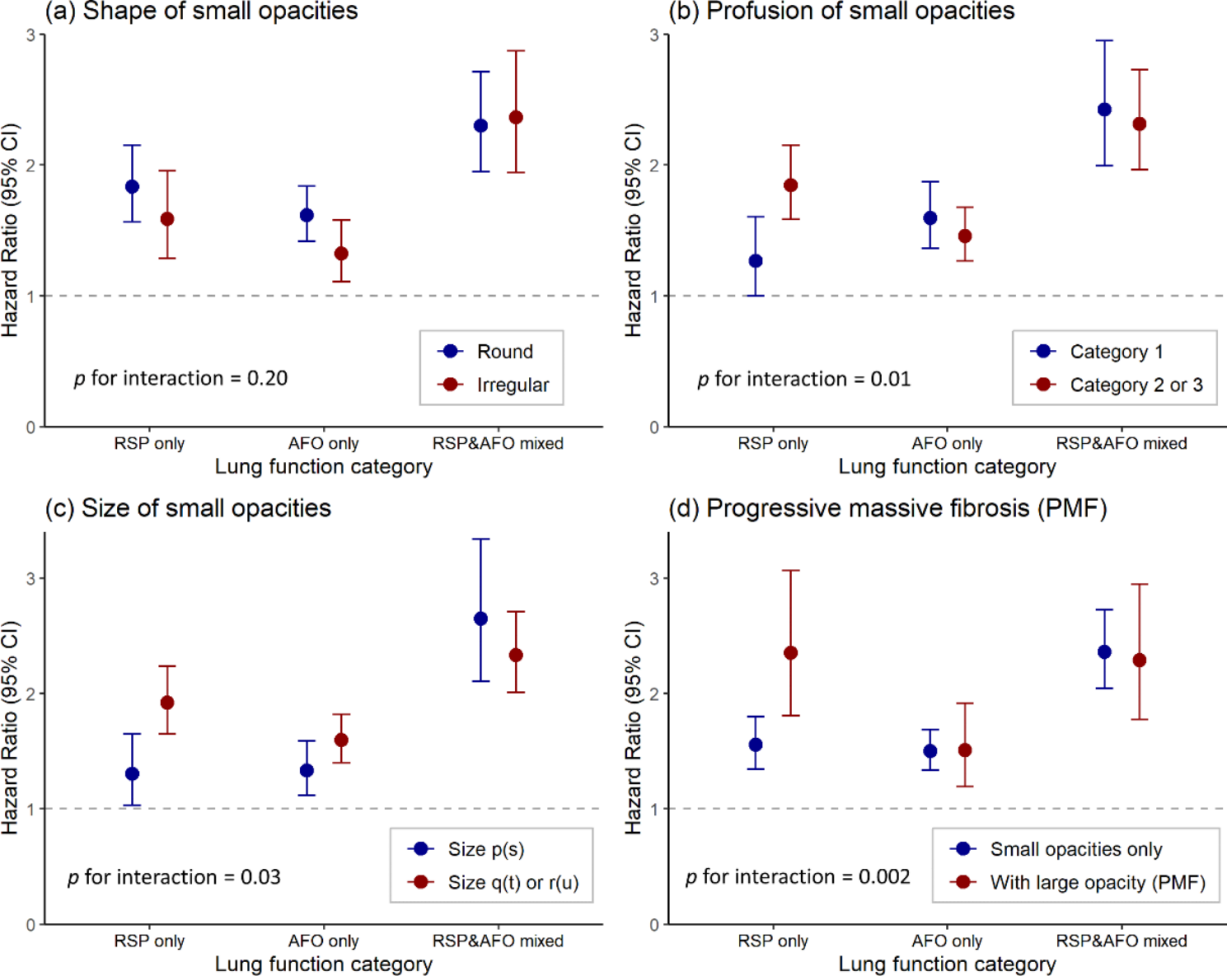
Association of spirometric restriction and airflow obstruction with all-cause mortality by (**a**) shape, (**b**) profusion, (**c**) size of small opacities and (**d**) progressive massive fibrosis (PMF) All hazard ratios were adjusted for age, BMI, smoking status, pack-years, tuberculosis, and cumulative silica exposure. Subjects with normal spirometry were used as reference Abbreviations: RSP, restrictive spirometry pattern; AFO, airflow obstruction; PMF, progressive massive fibrosis

The association of RSP with all-cause mortality differed between subjects with different extent of fibrotic changes in lungs, e.g., size and profusion of small opacities and progressive massive fibrosis (*p* for interaction < 0.05). In fact, silicotic nodules caused by fibrosis was a predictor of all-cause mortality independent of RSP and other covariates (Table E3). Subgroup analyses in groups split by radiographic characteristics of silicotic nodules revealed that the excess risk of all-cause mortality associated with RSP only was higher in subjects with large opacity (progressive massive fibrosis) or small opacities of larger size or higher profusion categories, but the risk of mortality did not differ significantly among subjects with round or irregular small opacities **(**Fig. 2, Table E4-E7). However, the excess risk caused by AFO only and RSP&AFO mixed did not differ significantly among subjects with different extent of fibrotic changes in lungs.

### Combined effect of RSP and smoking on all-cause mortality

**Fig. 3:**
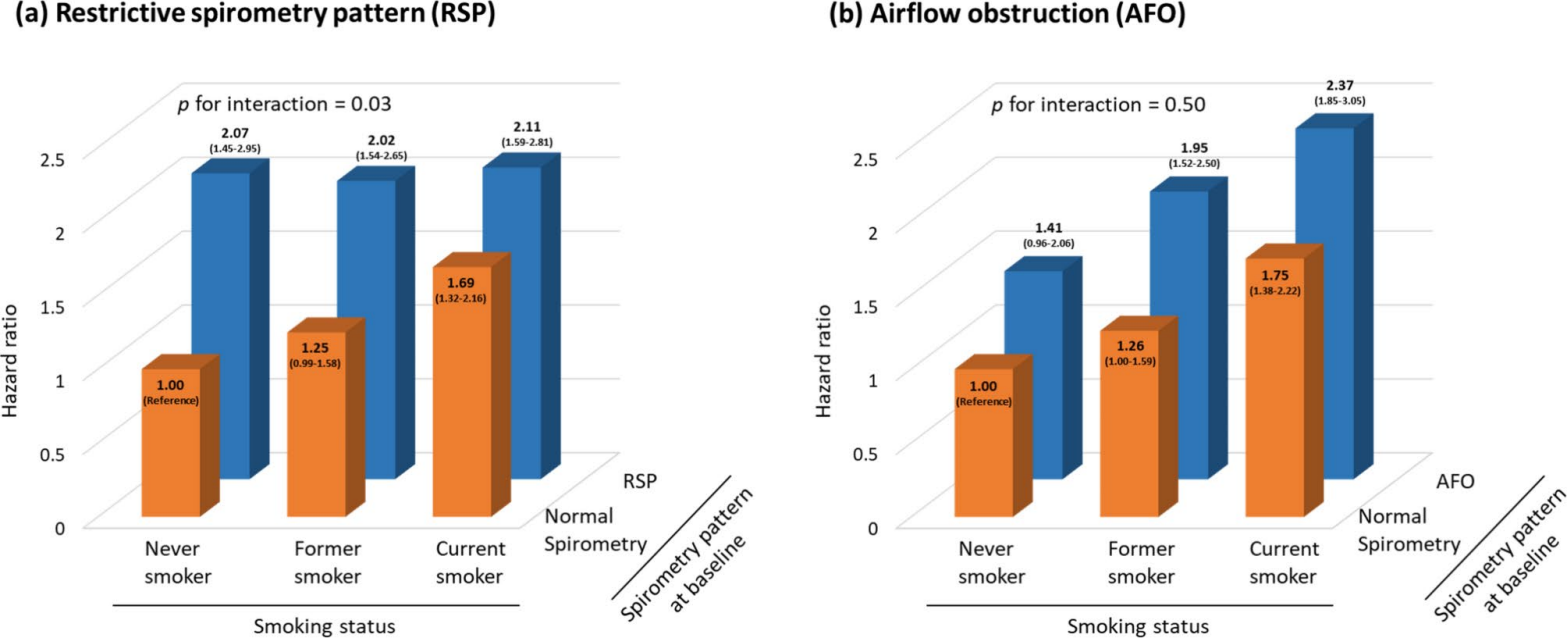
The combined effect of smoking with (**a**) restrictive spirometry pattern and (**b**) airflow obstruction on all-cause death The hazard ratios of all-cause mortality by smoking and spirometric restriction (**a**) were estimated in 2602 subjects without airflow obstruction (FEV_1_/FVC ≥ 0.70) at baseline and adjusted for age, BMI, tuberculosis, cumulative silica exposure, smoking pack-years, characteristics of small opacities (shape, size, profusion), and progressive massive fibrosis. The hazard ratio of all-cause mortality by smoking and airflow obstruction (**b**) was estimated in 3277 subjects without spirometric restriction (FVC < 80% predicted) at baseline and adjusted for age, BMI, tuberculosis, cumulative silica exposure, smoking pack-years, characteristics of small opacities (shape, size, profusion), and progressive massive fibrosis. Never smokers with normal spirometry at baseline were used as reference. Data are presented as the hazard ratio with 95% confidence interval Abbreviations: RSP, restrictive spirometry pattern; AFO, airflow obstruction; BMI, body mass index

RSP and smoking interacted significantly for the association with all-cause mortality (*p* for interaction = 0.03). Compared with the reference group (i.e., never smokers with normal spirometry), current smoking silicotics with normal spirometry showed an increased risk of all-cause mortality (HR = 1.69, 95% CI 1.32–2.16), but silicotics with RSP had similar risks no matter what the status of smoking was (never smoker: HR = 2.07, 95% CI 1.45–2.95; former smoker: HR = 2.02, 95% CI 1.54–2.65; current smoker: HR = 2.11, 95% CI 1.59–2.81) **(**Fig. 3a**)**. In contrast, AFO tended to increase the risk of all-cause mortality with no evidence of interaction with smoking status (*p* for interaction = 0.50) **(**Fig. 3b**)**.

### Sensitivity analyses

A total of 119 (2.8%) subjects were defined as non-RSP PRISm in the sensitivity analyses (Table E8). The mean age of this group was 50.2 (± 7.5), which was younger than other groups. Compared with subjects with normal spirometry, those with non-RSP PRISm had increased risk of both all-cause and spirometry-related mortality (all-cause mortality: crude HR = 1.42, 95% CI 1.12–1.79; respiratory-related mortality: crude HR = 1.92, 95% CI 1.47–2.51), and these associations remained significant after adjusting for age, body mass index, tuberculosis history, cumulative silica exposure, smoking status, pack-years, and radiographic changes (all-cause mortality: adjusted HR = 1.32, 95% CI 1.04–1.67; respiratory-related mortality: adjusted HR = 1.63, 95% CI 1.24–2.14) (Table E9). Besides, the LLN-defined RSP was also significantly associated with increased risk of all-cause and respiratory-related mortality (Table E10&E11), which was in accordance with the results based on fixed cut-off points of 0.7 (for FEV_1_/FVC ratio) and 80% (for FVC % predicted).

## Discussion

This large historical cohort study with up to 39 years of follow-up is the first to characterize the risk profile of RSP among patients with silicosis. Compared with silicotic patients with normal spirometry, those with solely RSP or mixed defect had 63% and 122% increased risk of all-cause mortality, respectively. Of note, there was a significant interaction between characteristics of lung opacities caused by silicosis and RSP in the association with all-cause mortality, showing that advanced fibrotic changes imposed additional risks of all-cause mortality in the silicotics with RSP. Moreover, this study suggested that the excess risk of all-cause death caused by RSP was not altered by smoking status, whilst silicotics with normal spirometry or AFO benefited from smoking cessation for a reduced risk of mortality.

In this study, we found that 23.1% of the 4315 subjects had RSP (11.0% for single RSP and 13.1% for mixed RSP&AFO, respectively) at the diagnosis of silicosis, which is higher than the prevalence of RSP ranging from 7.1 to 20.3% in the general population of a variety of countries with different incomes [10, 17, 18, 20, 22, 33–39]. Our finding of a 63% increased risk of all-cause mortality in the silicotic patients with RSP only was similar to the reports of previous population-based cohort studies in the United States [10, 20, 21, 35, 37, 39], Japan [17], Netherlands [18], China [22], United Kingdom [33], Italy [36], and Denmark [40]. These results were validated in the sensitivity analysis that adopted LLN-defined spirometry impairment. Subgroup analyses by chest radiographic features confirmed the robustness of this association and revealed higher risk of all-cause death for subjects with RSP in silicotics with advanced silicotic fibrosis, which is probably attributed to the higher proportion of true pulmonary restriction caused by fibrosis in this group. Our finding of significant correlation between the degree of fibrosis and FVC % predicted also suggested that the restrictive pattern and degree of restriction in silicosis might be a reflection of the extent of underlying lung lesion [41]. Since RSP was alternatively defined as low FEV_1_ with preserved FEV_1_/FVC and named PRISm in recent studies, we defined non-RSP PRISm in the sensitivity analyses and observed significantly increased risk of all-cause mortality in this group, indicating low FEV_1_ may have prognostic value in patients with silicosis.

The association of RSP with cause-specific mortality in the silicotics was not completely consistent with that in general population. In accordance with the findings from a US general population-based pooled cohort study [21], our study provided supportive evidence on a significant association of RSP with mortality from non-malignant respiratory diseases, the first leading cause of death in the silicotics. However, despite a significant association of RSP with cardiovascular-related mortality that has been previously reported in general population [10, 18], we did not observe a significantly increased risk of cardiovascular-related mortality in the silicotics with RSP. The reason might be the “healthy worker” effect that dust-exposed workers generally engaging in construction or tunnelling often exhibit lower risk of cardiovascular diseases due to their more physical fitness and better cardiopulmonary function than the general population [42, 43]. These results suggest that the early intervention on RSP may help in preventing the respiratory-related premature death, the leading threat of the silitotics, and thereby prolong the overall survival of this population.

In addition to the single RSP, we demonstrated a mixed restrictive and obstructive ventilatory defect among 13.1% of the entire cohort and 33.0% of the subjects with AFO, which is comparable with the prevalence that Kouranos et al. [44] previously reported in a large sarcoidosis cohort. However, this spirometry pattern has largely been overlooked and categorized as obstructive in previous population-based studies because of its low prevalence of around 3.5% in general population [45]. A possible reason for such a high prevalence of the mixed defect in the silicotics is the airway involvement caused by pulmonary fibrosis. In fibrotic lung diseases like silicosis, pulmonary fibrosis is not only a clinical cause of restrictive lung defects but also can lead to airway distortion, which may cause airway obstruction [46]. Our study also observed notably higher risk of both all-cause and respiratory-related mortality in the silicotics with RSP&AFO mixed than in those with single RSP or AFO, which is probably attributed to the increased incidence of concomitant pulmonary hypertension [47]. Although a mixed restrictive and obstructive defect is of low prevalence in general population, these findings highlighted the necessity of subdividing this spirometry pattern in the patients with silicosis.

Smoking has been determined as an independent predictor of all-cause mortality in several population-based studies [48–50]. Our study observed higher risk of all-cause mortality in current smokers than that in former smokers and never smokers in the silicotics with normal spirometry and AFO, suggesting the health benefit of smoking cessation and emphasizing the importance of anti-tobacco campaign among this population. However, the risk remained unchanged in RSP no matter whether they smoked or not, indicating that RSP may mask the effect of smoking in the silicosis population. These findings suggest further research on the pathophysiological mechanism of smoking among the silicotics with RSP who possesses heavier smoking and earlier initiation of smoking than the general population [24, 51].

The strengths of this study include a large sample size with a territory-wide coverage of study population, high-quality spirometry data, a long period of follow-up over 39 years with a high follow-up rate, and the integrity of data on radiographic characteristics, lifelong smoking habits, and verified survival outcomes. However, some potential limitations should be considered. Firstly, our findings were based on the pre-bronchodilator spirometry data, which may lead to the misclassification of asthma. However, this should be of less importance because our major focus was on the subjects with a preserved ratio of FEV_1_/FVC instead of those with obstructive lung defects such as COPD. Secondly, the cumulative silica exposure was estimated using JEM rather than directly measured by individual-level personal monitoring. However, JEM has been widely adopted to assess the occupational exposures in epidemiological studies with satisfying power of exposure estimation [52, 53], thus it is less likely to be a risk of residual confounding. The consistent results from the sensitivity analysis adjusting for the duration of silica exposure rather than cumulative silica exposure estimated using JEM supported this. Thirdly, some unmeasured predictors, e.g., ambient/indoor air pollution, physical activities [54, 55], diets [56–58], shift work [59], and alcohol intake [60], are also associated with mortality but were not controlled in the current study. Therefore, further study including these risk factors are warranted.

## Conclusions

The present study provided evidence that RSP is associated with increased all-cause and respiratory-related mortality in patients with silicosis, indicating that RSP accounts for a fraction of extra death. To prolong the lifespan of silicotics and improve their quality of life, RSP, especiallymixed ventilatory defect of RSP in combination of AFO, shall be recognized and managed properly in the occupational settings.

### Electronic supplementary material

Below is the link to the electronic supplementary material.

Supplementary Material 1

## Data Availability

The participants of this study did not give written consent for their data to be shared publicly, so due to the sensitive nature of the research supporting data is not available but it can be available from the corresponding author on reasonable request and with the permission of Hong Kong Department of Health.
