# Supplementary material for: Association of spirometric restriction with mortality in the silicotics: a cohort study
Source: BMC Pulm Med. 2023 Sep 4;23:327. doi: 10.1186/s12890-023-02622-1 (PMC10478203; doi:10.1186/s12890-023-02622-1)
Supplement: Supplementary file 1 — Supplementary Material 1 [file 12890_2023_2622_MOESM1_ESM.pdf]

ONLINE DATA SUPPLEMENT

Association of spirometric restriction with mortality in silicotics: a cohort study

Shuyuan Yang, Chi Kuen Chan, Maggie Haitian Wang, Chi Chiu Leung, Lai Bun Tai, Lap Ah Tse.

Table E1. Inclusion and exclusion of the study subjects

Study population	Subjects No. (%)
Excluded subjects	166 (3.7%)
> 80 years old#	83 (1.9%)
No record of baseline examination	28 (0.6%)
Invalid spirometry data*	55 (1.2%)
Included subjects	4315 (96.3%)
Total	4481 (100.0%)

# Prediction formulae for reference values and LLNs are applicable for subjects aged 18-80 years, thus those aged over 80 years were excluded[1].

* The spirometry results were considered as invalid when (1) FEV_1: < 0.2 L/s or > 7.0 L/s; (2) FEV_1 % predicted: < 10% or > 140%; (3) FVC: < 0.2 L or > 7.0 L; (4) FVC % predicted: < 10% or > 140%; or (5) FEV_1/FVC ratio: < 0.1 or > 1.0[2]

[1] Ip MS, Ko FW, Lau AC, et al. Updated spirometric reference values for adult Chinese in Hong Kong and implications on clinical utilization. *Chest.* 2006;129(2):384-392. doi:10.1378/chest.129.2.384

[2] Josephs L, Culliford D, Johnson M, Thomas M. Improved outcomes in ex-smokers with COPD: a UK primary care observational cohort study. *Eur Respir J.* 2017;49(5):1602114. Published 2017 May 23. doi:10.1183/13993003.02114-2016

Table E2. Number and frequencies of underlying cause of death in the cohort during 1981-2019

Cause of death	ICD-10 code	Death No. (%)
Certain infectious and parasitic diseases	A00-B99	41 (1.7)
Malignant neoplasms	C00-C97	513 (21.4)
Other neoplasms	D00-D48	2 (0.1)
Diseases of the blood and blood-forming organs and certain disorders involving the immune mechanism	D50-D89	4 (0.2)
Endocrine, nutritional, and metabolic diseases	E00-E90	18 (0.8)
Mental and behavioral disorders	F00-F99	4 (0.2)
Diseases of the nervous system	G00-G99	3 (0.1)
Diseases of the eye and adnexa	H00-H59	2 (0.1)
Diseases of the circulatory system	I00-I99	168 (7.0)
Diseases of the respiratory system	J00-J99	1359 (56.6)
Diseases of the digestive system	K00-K93	45 (1.9)
Diseases of the skin and subcutaneous tissue	L00-L99	1 (0.0)
Diseases of the musculoskeletal system and connective tissue	M00-M99	5 (0.2)
Diseases of the genitourinary system	N00-N99	20 (0.8)
Congenital malformations, deformations, and chromosomal abnormalities	Q00-Q99	2 (0.1)
Symptoms, signs, and abnormal clinical and laboratory findings, not elsewhere classified	R00-R99	56 (2.3)
External causes of morbidity and mortality	V01-Y98	156 (6.5)
Total	A00-Z99	2399 (100.0)

Table E3. Hazard ratios (HR) and 95% confidence intervals (CI) for all-cause mortality by radiographic characteristics of lung opacities

Radiographic characteristics	No. of deaths/ subjects	Crude incidence rate*	Univariate		Multivariate#	
			HR (95% CI)	*p*-value	HR (95% CI)	*p*-value
Shape of small nodules						
Round	1497/2873	40.0	1.00 (Ref.)		1.00 (Ref.)	
Irregular	869/1393	46.4	1.10 (1.01, 1.20)	0.03	0.97 (0.89, 1.06)	0.55
Size of small nodules						
Category p or s	825/1796	37.3	1.00 (Ref.)		1.00 (Ref.)	
Category q or t	1266/2092	42.7	1.16 (1.06, 1.26)	0.001	1.02 (0.93, 1.12)	0.66
Category r or u	275/378	63.7	1.89 (1.65, 2.17)	<0.001	1.48 (1.29, 1.70)	<0.001
Profusion of small nodules						
Category 1 (1/0, 1/1, 1/2)	980/2384	35.0	1.00 (Ref.)		1.00 (Ref.)	
Category 2 (2/1, 2/2, 2/3)	1092/1528	46.9	1.23 (1.12, 1.34)	<0.001	1.16 (1.07, 1.27)	<0.001
Category 3 (3/2, 3/3, 3/+)	293/350	62.1	1.66 (1.46, 1.90)	<0.001	1.76 (1.54, 2.02)	<0.001
Progressive massive fibrosis						
No (small opacities only)	1846/3516	39.3	1.00 (Ref.)		1.00 (Ref.)	
Yes (with large opacity)	531/763	57.1	1.58 (1.43, 1.74)	<0.001	1.29 (1.16, 1.42)	<0.001
Size of large opacity						
Category A	354/503	52.6	1.00 (Ref.)		1.00 (Ref.)	
Category B	135/208	61.5	1.27 (1.04, 1.55)	0.02	1.30 (1.06, 1.60)	0.01
Category C	42/48	119.1	3.06 (2.21, 4.24)	<0.001	2.16 (1.53, 3.06)	<0.001

Abbreviations: HR, hazard ratio; CI, confidence interval.

* Per 10^3 person-years.

# Adjusted for age, BMI category, history of tuberculosis, cumulative silica exposure, smoking status, pack-years, and lung function category.

Table E4. Subgroup analyses of the association of baseline lung function categories with risk of all-cause mortality by shape of small opacities

Lung function category	No. of deaths/ subjects	Crude mortality rate*	Crude HR (95% CI)	Adjusted HR (95% CI)		
			Model 0	Model 1	Model 2	Model 3
Analysis for subjects with small round opacities (n=2873)						
Normal spirometry	650/1483	29.6	1.00 (Ref.)	1.00 (Ref.)	1.00 (Ref.)	1.00 (Ref.)
RSP only	206/290	55.2	1.94 (1.66, 2.28)	1.83 (1.56, 2.14)	1.81 (1.54, 2.12)	1.84 (1.57, 2.15)
AFO only	427/802	48.6	2.04 (1.80, 2.31)	1.70 (1.50, 1.94)	1.68 (1.48, 1.92)	1.62 (1.42, 1.84)
RSP&AFO mixed	214/298	73.7	3.11 (2.66, 3.64)	2.48 (2.11, 2.91)	2.40 (2.04, 2.83)	2.30 (1.95, 2.71)
Analysis for subjects with small irregular opacities (n=1393)						
Normal spirometry	329/628	32.9	1.00 (Ref.)	1.00 (Ref.)	1.00 (Ref.)	1.00 (Ref.)
RSP only	124/176	54.5	1.75 (1.43, 2.16)	1.62 (1.32, 2.00)	1.61 (1.30, 1.98)	1.59 (1.29, 1.96)
AFO only	230/335	52.4	1.79 (1.51, 2.12)	1.40 (1.17, 1.67)	1.37 (1.15, 1.64)	1.32 (1.11, 1.58)
RSP&AFO mixed	186/254	89.6	3.46 (2.88, 4.15)	2.55 (2.10, 3.09)	2.51 (2.07, 3.04)	2.37 (1.95, 2.87)

Abbreviations: AFO, airflow obstruction; PRISm, preserved ratio impaired spirometry; RSP, restrictive spirometry pattern; HR, hazard ratio; CI, confidence interval.

Model 0: no adjustments.

Model 1: adjusted for age and BMI category.

Model 2: adjusted for the covariates in Model 1 plus history of tuberculosis, and cumulative silica exposure.

Model 3: adjusted for the covariates in Model 2 plus smoking status and pack-years.

* Per 10^3 person-years.

Table E5. Subgroup analyses of the association of baseline lung function categories with risk of all-cause mortality by profusion of small opacities

Lung function category	No. of deaths/ subjects	Crude mortality rate*	Crude HR (95% CI)	Adjusted HR (95% CI)		
			Model 0	Model 1	Model 2	Model 3
Analysis for subjects with profusion of small opacities in Category 1 (n=2384)						
Normal spirometry	432/1284	25.2	1.00 (Ref.)	1.00 (Ref.)	1.00 (Ref.)	1.00 (Ref.)
RSP only	85/162	41.2	1.60 (1.26, 2.01)	1.30 (1.03, 1.65)	1.29 (1.02, 1.64)	1.27 (1.00, 1.61)
AFO only	303/674	45.3	2.28 (1.96, 2.65)	1.71 (1.46, 2.00)	1.69 (1.44, 1.97)	1.60 (1.36, 1.87)
RSP&AFO mixed	160/264	76.3	3.73 (3.11, 4.49)	2.61 (2.15, 3.16)	2.56 (2.11, 3.11)	2.43 (2.00, 2.95)
Analysis for subjects with profusion of small opacities in Category 2 (n=1528)						
Normal spirometry	458/719	35.3	1.00 (Ref.)	1.00 (Ref.)	1.00 (Ref.)	1.00 (Ref.)
RSP only	166/217	56.4	1.79 (1.50, 2.14)	1.70 (1.42, 2.04)	1.70 (1.42, 2.04)	1.76 (1.47, 2.11)
AFO only	290/382	54.3	1.81 (1.56, 2.10)	1.53 (1.32, 1.79)	1.53 (1.31, 1.78)	1.50 (1.28, 1.75)
RSP&AFO mixed	178/210	87.8	3.45 (2.89, 4.12)	2.77 (2.30, 3.33)	2.74 (2.28, 3.30)	2.67 (2.21, 3.21)
Analysis for subjects with profusion of small opacities in Category 3 (n=350)						
Normal spirometry	88/107	48.4	1.00 (Ref.)	1.00 (Ref.)	1.00 (Ref.)	1.00 (Ref.)
RSP only	79/88	78.8	1.77 (1.30, 2.40)	1.74 (1.27, 2.38)	1.68 (1.23, 2.31)	1.50 (1.08, 2.08)
AFO only	64/79	59.3	1.31 (0.95, 1.82)	1.21 (0.87, 1.69)	1.26 (0.90, 1.77)	1.14 (0.81, 1.61)
RSP&AFO mixed	62/76	75.8	1.84 (1.32, 2.56)	1.42 (1.00, 2.01)	1.35 (0.94, 1.92)	1.15 (0.80, 1.66)

Abbreviations: AFO, airflow obstruction; PRISm, preserved ratio impaired spirometry; RSP, restrictive spirometry pattern; HR, hazard ratio; CI, confidence interval.

Model 0: no adjustments.

Model 1: adjusted for age and BMI category.

Model 2: adjusted for the covariates in Model 1 plus history of tuberculosis, and cumulative silica exposure.

Model 3: adjusted for the covariates in Model 2 plus smoking status and pack-years.

* Per 10^3 person-years.

Table E6. Subgroup analyses of the association of baseline lung function categories with risk of all-cause mortality by size of small opacities

Lung function category	No. of deaths/ subjects	Crude mortality rate*	Crude HR (95% CI)	Adjusted HR (95% CI)		
			Model 0	Model 1	Model 2	Model 3
Analysis for subjects with small opacities in size p(s) (n=1796)						
Normal spirometry	403/984	29.3	1.00 (Ref.)	1.00 (Ref.)	1.00 (Ref.)	1.00 (Ref.)
RSP only	89/152	44.8	1.59 (1.27, 2.00)	1.35 (1.07, 1.71)	1.33 (1.05, 1.68)	1.30 (1.03, 1.65)
AFO only	231/485	45.2	1.95 (1.65, 2.30)	1.44 (1.21, 1.71)	1.39 (1.17, 1.66)	1.33 (1.12, 1.59)
RSP&AFO mixed	102/175	80.3	3.69 (2.96, 4.60)	2.72 (2.16, 3.41)	2.75 (2.19, 3.46)	2.65 (2.10, 3.34)
Analysis for subjects with small opacities in size q(t) (n=2092)						
Normal spirometry	509/1022	30.5	1.00 (Ref.)	1.00 (Ref.)	1.00 (Ref.)	1.00 (Ref.)
RSP only	191/256	56.9	1.98 (1.68, 2.34)	1.89 (1.60, 2.23)	1.88 (1.59, 2.23)	1.91 (1.61, 2.26)
AFO only	343/531	51.2	1.94 (1.69, 2.23)	1.63 (1.41, 1.88)	1.61 (1.40, 1.86)	1.57 (1.36, 1.81)
RSP&AFO mixed	223/283	75.5	3.03 (2.58, 3.55)	2.41 (2.04, 2.84)	2.36 (2.00, 2.79)	2.27 (1.92, 2.69)
Analysis for subjects with small opacities in size r(u) (n=378)						
Normal spirometry	67/105	43.8	1.00 (Ref.)	1.00 (Ref.)	1.00 (Ref.)	1.00 (Ref.)
RSP only	50/58	75.0	1.66 (1.15, 2.42)	1.69 (1.15, 2.47)	1.71 (1.16, 2.51)	1.71 (1.15, 2.53)
AFO only	83/121	60.7	1.53 (1.10, 2.12)	1.46 (1.05, 2.03)	1.54 (1.10, 2.15)	1.57 (1.12, 2.22)
RSP&AFO mixed	75/94	99.2	2.70 (1.93, 3.79)	2.27 (1.60, 3.22)	2.35 (1.65, 3.36)	2.35 (1.63, 3.39)

Abbreviations: AFO, airflow obstruction; PRISm, preserved ratio impaired spirometry; RSP, restrictive spirometry pattern; HR, hazard ratio; CI, confidence interval.

Model 0: no adjustments.

Model 1: adjusted for age and BMI category.

Model 2: adjusted for the covariates in Model 1 plus history of tuberculosis, and cumulative silica exposure.

Model 3: adjusted for the covariates in Model 2 plus smoking status and pack-years.

* Per 10^3 person-years.

Table E7. Subgroup analyses of the association of baseline lung function categories with risk of all-cause mortality by progressive massive fibrosis

Lung function category	No. of deaths/ subjects	Crude mortality rate*	Crude HR (95% CI)	Adjusted HR (95% CI)		
			Model 0	Model 1	Model 2	Model 3
Analysis for subjects with small opacities only (without PMF) (n=3516)						
Normal spirometry	822/1853	29.3	1.00 (Ref.)	1.00 (Ref.)	1.00 (Ref.)	1.00 (Ref.)
RSP only	239/352	49.6	1.72 (1.49, 1.99)	1.56 (1.35, 1.81)	1.54 (1.33, 1.78)	1.56 (1.34, 1.80)
AFO only	499/900	47.9	1.94 (1.74, 2.18)	1.58 (1.41, 1.78)	1.56 (1.39, 1.75)	1.50 (1.34, 1.69)
RSP&AFO mixed	286/411	76.7	3.32 (2.90, 3.80)	2.53 (2.19, 2.91)	2.48 (2.15, 2.86)	2.36 (2.04, 2.73)
Analysis for subjects with large opacity (with PMF) (n=763)						
Normal spirometry	158/259	40.0	1.00 (Ref.)	1.00 (Ref.)	1.00 (Ref.)	1.00 (Ref.)
RSP only	93/117	76.9	2.28 (1.76, 2.96)	2.37 (1.82, 3.09)	2.37 (1.82, 3.08)	2.35 (1.81, 3.06)
AFO only	161/241	57.2	1.73 (1.38, 2.17)	1.48 (1.17, 1.87)	1.48 (1.17, 1.87)	1.51 (1.19, 1.92)
RSP&AFO mixed	119/146	89.9	2.79 (2.19, 3.56)	2.38 (1.85, 3.06)	2.37 (1.84, 3.04)	2.29 (1.78, 2.95)

Abbreviations: AFO, airflow obstruction; PRISm, preserved ratio impaired spirometry; RSP, restrictive spirometry pattern; PMF, progressive massive fibrosis; HR, hazard ratio; CI, confidence interval.

Model 0: no adjustments.

Model 1: adjusted for age and BMI category.

Model 2: adjusted for the covariates in Model 1 plus history of tuberculosis, and cumulative silica exposure.

Model 3: adjusted for the covariates in Model 2 plus smoking status and pack-years.

* Per 10^3 person-years.

Table E8. Distribution of subjects and characteristics of lung function by spirometry patterns

Spirometry pattern			Subject No. (%)	Lung function category	Age	FEV_1 % predicted	FVC % predicted	FEV_1/FVC ratio
FEV_1/FVC	FEV_1	FVC						
Non-obstructive	Normal	Normal	2010 (46.6)	Normal spirometry	53.4±9.8	99.7±11.7	100.6±11.3	0.79±0.06
Non-obstructive	Low	Normal	119 (2.8)	Normal spirometry/Non-RSP PRISm*	50.2±7.5	76.5±2.3	84.4±3.1	0.73±0.03
Non-obstructive	Normal	Low	84 (1.9)	RSP only	57.9±11.1	84.5±4.2	75.3±3.8	0.88±0.06
Non-obstructive	Low	Low	389 (9.0)	RSP only	55.5±9.6	65.0±11.6	65.3±12.3	0.79±0.07
Obstructive	Normal	Normal	435 (10.1)	AFO only	59.9±9.6	91.0±8.5	108.7±11.0	0.65±0.04
Obstructive	Low	Normal	713 (16.5)	AFO only	59.8±9.4	65.2±11.7	92.2±9.0	0.55±0.10
Obstructive	Normal	Low	0 (0.0)	NA	NA	NA	NA	NA
Obstructive	Low	Low	565 (13.1)	RSP&AFO mixed	60.5±9.7	44.4±13.6	66.1±11.3	0.52±0.12

Abbreviations: AFO, airflow obstruction; PRISm, preserved ratio impaired spirometry; RSP, restrictive spirometry pattern; BMI, body mass index.
Non-obstructive: FEV1/FVC ≥ 0.70; Obstructive: FEV1/FVC < 0.70; Normal: ≥ 80% predicted; Low: < 80% predicted. Data are presented as mean±SD.
* In sensitivity analysis.

Table E9. Hazard ratios (HR) and 95% confidence intervals (CI) for all-cause and major cause-specific mortality by lung function categories with Non-RSP PRISm

Lung function category	No. of deaths/ subjects	Crude mortality rate*	Crude HR (95% CI)	Adjusted HR (95% CI)		
			Model 0	Model 1	Model 2	Model 3
All-cause mortality						
Normal spirometry	915/2010	30.0	1.00 (Ref.)	1.00 (Ref.)	1.00 (Ref.)	1.00 (Ref.)
Non-RSP PRISm	76/119	40.8	1.42 (1.12, 1.79)	1.42 (1.13, 1.80)	1.40 (1.10, 1.77)	1.32 (1.04, 1.67)
RSP only	335/473	55.5	1.95 (1.72, 2.21)	1.79 (1.58, 2.03)	1.80 (1.59, 2.04)	1.67 (1.46, 1.89)
AFO only	662/1148	49.6	1.98 (1.79, 2.19)	1.59 (1.43, 1.76)	1.54 (1.38, 1.71)	1.50 (1.34, 1.67)
RSP&AFO mixed	411/565	80.0	3.39 (3.01, 3.81)	2.56 (2.26, 2.89)	2.43 (2.15, 2.75)	2.26 (1.99, 2.57)
Respiratory-related mortality						
Normal spirometry	448/2010	14.7	1.00 (Ref.)	1.00 (Ref.)	1.00 (Ref.)	1.00 (Ref.)
Non-RSP PRISm	51/119	27.4	1.92 (1.47, 2.51)	1.79 (1.37, 2.33)	1.78 (1.36, 2.32)	1.63 (1.24, 2.14)
RSP only	193/473	32.0	1.97 (1.66, 2.34)	1.84 (1.55, 2.18)	1.84 (1.55, 2.19)	1.63 (1.37, 1.94)
AFO only	379/1148	28.4	1.86 (1.63, 2.12)	1.70 (1.48, 1.95)	1.68 (1.46, 1.94)	1.61 (1.40, 1.86)
RSP&AFO mixed	288/565	56.1	3.56 (3.05, 4.15)	3.08 (2.62, 3.63)	3.03 (2.57, 3.58)	2.70 (2.27, 3.21)

Abbreviations: AFO, airflow obstruction; PRISm, preserved ratio impaired spirometry; RSP, restrictive spirometry pattern; HR, hazard ratio; CI, confidence interval.

Model 0: no adjustments.

Model 1: adjusted for age, BMI category, history of tuberculosis, and cumulative silica exposure.

Model 2: adjusted for the covariates in Model 1 plus smoking status and pack-years.

Model 3: adjusted for the covariates in Model 2 plus the radiographic characteristics of the silicotic nodules, including shape, size, profusion of the small opacities and progressive massive fibrosis.

* Per 10^3 person-years.

Table E10. Distribution of subjects and characteristics of lung function by LLN-defined spirometry patterns

Spirometry pattern (defined by LLN)			Subject No. (%)	Lung function category	Age	FEV_1 % predicted	FVC % predicted	FEV_1/FVC ratio
FEV_1/FVC	FEV_1	FVC						
Non-obstructive	Normal	Normal	2182 (50.6)	Normal spirometry - LLN	55.2±10.2	98.4±12.4	99.4±12	0.79±0.07
Non-obstructive	Low	Normal	139 (3.2)	Normal spirometry - LLN	62.0±9.7	72.8±3.6	81.5±4.3	0.69±0.05
Non-obstructive	Normal	Low	68 (1.6)	RSP only - LLN	59.1±10.3	80.9±4.7	72.0±4.1	0.88±0.06
Non-obstructive	Low	Low	320 (7.4)	RSP only - LLN	58.8±10.4	61.9±11.1	62.8±12.1	0.78±0.08
Obstructive	Normal	Normal	527 (12.2)	AFO only - LLN	52.3±9.1	87.4±9.2	106.4±11.4	0.66±0.06
Obstructive	Low	Normal	666 (15.4)	AFO only - LLN	58.8±9.3	60.2±12.1	89.0±9.6	0.53±0.11
Obstructive	Normal	Low	0 (0.0)	NA	NA	NA	NA	NA
Obstructive	Low	Low	413 (9.6)	RSP&AFO mixed - LLN	58.4±9.8	40.8±12.6	62.4±10.9	0.51±0.13

Abbreviations: AFO, airflow obstruction; PRISm, preserved ratio impaired spirometry; RSP, restrictive spirometry pattern; LLN, lower limit of normal.

Non-obstructive: FEV_1/FVC ≥ LLN; Obstructive: FEV_1/FVC < LLN; Normal: ≥ LLN; Low: < LLN. Data are presented as mean±SD.

Table E11. Hazard ratios (HR) and 95% confidence intervals (CI) for all-cause and cause-specific mortality by LLN-defined lung function categories

Lung function category	No. of deaths/ subjects	Crude mortality rate*	Crude HR (95% CI)	Adjusted HR (95% CI)		
			Model 0	Model 1	Model 2	Model 3
All-cause mortality						
Normal spirometry - LLN	1102/2321	33.2	1.00 (Ref.)	1.00 (Ref.)	1.00 (Ref.)	1.00 (Ref.)
RSP only - LLN	271/388	60.3	1.90 (1.67, 2.17)	1.70 (1.49, 1.94)	1.70 (1.49, 1.95)	1.60 (1.39, 1.83)
AFO only - LLN	713/1193	46.4	1.49 (1.35, 1.64)	1.45 (1.31, 1.59)	1.42 (1.29, 1.56)	1.39 (1.26, 1.53)
RSP&AFO mixed - LLN	313/413	82.4	2.92 (2.58, 3.32)	2.46 (2.16, 2.80)	2.32 (2.04, 2.65)	2.16 (1.88, 2.47)
Respiratory-related mortality						
Normal spirometry - LLN	535/2321	16.1	1.00 (Ref.)	1.00 (Ref.)	1.00 (Ref.)	1.00 (Ref.)
RSP only - LLN	163/388	36.2	2.08 (1.73, 2.50)	1.87 (1.55, 2.24)	1.85 (1.54, 2.23)	1.66 (1.37, 2.01)
AFO only - LLN	440/1193	28.7	1.79 (1.58, 2.02)	1.69 (1.50, 1.92)	1.68 (1.49, 1.91)	1.63 (1.44, 1.85)
RSP&AFO mixed - LLN	221/413	58.2	3.39 (2.86, 4.01)	2.89 (2.42, 3.45)	2.83 (2.37, 3.38)	2.47 (2.05, 2.99)
Lung cancer mortality						
Normal spirometry - LLN	140/2321	4.2	1.00 (Ref.)	1.00 (Ref.)	1.00 (Ref.)	1.00 (Ref.)
RSP only - LLN	26/388	5.8	1.04 (0.69, 1.59)	1.01 (0.66, 1.54)	1.08 (0.71, 1.66)	1.09 (0.71, 1.69)
AFO only - LLN	70/1193	4.6	0.95 (0.71, 1.26)	0.95 (0.71, 1.27)	0.87 (0.65, 1.17)	0.89 (0.66, 1.21)
RSP&AFO mixed - LLN	13/413	3.4	0.50 (0.28, 0.88)	0.46 (0.25, 0.83)	0.46 (0.26, 0.83)	0.46 (0.25, 0.84)
Cardiovascular-related mortality						
Normal spirometry - LLN	94/2321	2.8	1.00 (Ref.)	1.00 (Ref.)	1.00 (Ref.)	1.00 (Ref.)
RSP only - LLN	20/388	4.4	1.18 (0.73, 1.91)	1.16 (0.70, 1.92)	1.16 (0.69, 1.92)	1.20 (0.72, 2.00)
AFO only - LLN	36/1193	2.3	0.72 (0.49, 1.06)	0.76 (0.52, 1.13)	0.76 (0.52, 1.13)	0.77 (0.51, 1.15)
RSP&AFO mixed - LLN	18/413	4.7	1.03 (0.62, 1.71)	1.07 (0.63, 1.81)	1.09 (0.64, 1.86)	1.08 (0.62, 1.89)

Abbreviations: AFO, airflow obstruction; PRISm, preserved ratio impaired spirometry; RSP, restrictive spirometry pattern; HR, hazard ratio; CI, confidence interval.

Model 0: no adjustments.

Model 1: adjusted for age, BMI category, history of tuberculosis, and cumulative silica exposure.

Model 2: adjusted for the covariates in Model 1 plus smoking status and pack-years.

Model 3: adjusted for the covariates in Model 2 plus the radiographic characteristics of the silicotic nodules, including shape, size, profusion of the small opacities and progressive massive fibrosis.

* Per 10^3 person-years.
